# The Interrelationships between Intestinal Permeability and Phlegm Syndrome and Therapeutic Potential of Some Medicinal Herbs

**DOI:** 10.3390/biom11020284

**Published:** 2021-02-15

**Authors:** Junghyun Park, Tae Joon Choi, Ki Sung Kang, Seo-Hyung Choi

**Affiliations:** 1 College of Korean Medicine, Gachon University, Seongnam 13120, Korea; iwbstill@yahoo.com; 2 Wooje Research Institute for Integrative Medicine, WoojeIM, 432 Yeoksam-ro, Gangnam-gu, Seoul 06200, Korea; tjchoi@weedahm.com; 3 Department of Oriental Internal Medicine, Weedahm Oriental Hospital, 430 Yeoksam-ro, Gangnam-gu, Seoul 06200, Korea; 4 Department of Oriental Internal Medicine, Gangnam Weedahm Oriental Hospital, 402 Samsung-ro, Gangnam-gu, Seoul 06185, Korea

**Keywords:** increased GI barrier permeability, phlegm syndrome, inflammation, medicinal plants, phytochemicals, gut microbiome

## Abstract

The gastrointestinal (GI) tract has an intriguing and critical role beyond digestion in both modern and complementary and alternative medicine (CAM), as demonstrated by its link with the immune system. In this review, we attempted to explore the interrelationships between increased GI permeability and phlegm, an important pathological factor in CAM, syndrome, and therapeutic herbs for two disorders. The leaky gut and phlegm syndromes look considerably similar with respect to related symptoms, diseases, and suitable herbal treatment agents, including phytochemicals even though limitations to compare exist. Phlegm may be spread throughout the body along with other pathogens via the disruption of the GI barrier to cause several diseases sharing some parts of symptoms, diseases, and mechanisms with leaky gut syndrome. Both syndromes are related to inflammation and gut microbiota compositions. Well-designed future research should be conducted to verify the interrelationships for evidence based integrative medicine to contribute to the promotion of public health. In addition, systems biology approaches should be adopted to explore the complex synergistic effects of herbal medicine and phytochemicals on conditions associated with phlegm and leaky gut syndromes.

## 1. Introduction

The gastrointestinal (GI) tract has an intriguing and critical role beyond digestion, as demonstrated by its link with the immune system. In addition, the gut–brain axis theory describes the marvelous function of the GI tract, particularly of the gut microbiome. This axis is a bidirectional communication between the microbiota and the brain via a variety of routes such as the immune system providing the biological basis of neurodegenerative and psychiatric disorders. The GI barrier plays a crucial role in preventing the entry of toxins, chemicals, and pathogens into the blood stream via junctional complexes and helps maintain the integrity of the GI tract. A growing body of evidence indicates that increased intestinal permeability can serve as the causative factor in a wide range of diseases, including celiac disease, inflammatory bowel disease (IBD), type 1 diabetes, and obesity [1,2]. This state, referred to as “leaky gut”, which is a state of augmented GI permeability, may help explain the physiological process of pathogen entry into the blood stream along with several factors typically considered in TEAM, such as Phlegm, Spleen Qi, and Damp, using a holistic approach that has been used traditionally for several thousand years in theory as well as in clinical practice [3]. Since a “leaky gut” is directly related to inflammation in complementary and alternative medicine (CAM) [4], this could help bridge the philosophical and theoretical gap between the concepts used in TEAM and modern biomedicine [3]. Of the several factors considered important in TEAM, Phlegm is an endogenous pathological agent that can accumulate in any part of the body and cause diverse diseases, including dysfunction of the Spleen–Stomach (a part of the digestive system and the sources of Qi and blood in TEAM). Such dysfunction may cause GI disturbances, because in addition to playing a role in the production and flow of blood, the spleen is also considered to be associated with other organs of the digestive system, including the stomach and small or large intestines, in TEAM.

Therefore, the authors have reviewed the role of the GI barrier, definition of Phlegm syndrome, the interrelationships among leaky gut, Phlegm, and inflammation and impact on gut microbiota. Furthermore, we have also tried to compare diseases related to the leaky gut syndrome and Phlegm syndrome and traditional medicinal plants used for treating these diseases in CAM based on evidence from the current literature even though there are limitations for the comparison. Lastly, we assumed that Phlegm may invade the blood stream along with other pathogens via the dysfunction of the GI barrier, which is responsible for various diseases.

## 2. Study Design and Search Approach

We adopted a literature review to explore this topic [5]. A search was performed for English and Korean literature in online databases, including PubMed, Google Scholar, and ScienceDirect. Several keywords, such as “increased GI barrier permeability”, “intestinal barrier”, “leaky gut”, “phlegm syndrome”, “intestinal microbiota”, and “inflammation”, were used. 

In order to distinguish between terms used in Western medicine and traditional East Asian medicine (TEAM), the first letters of the terms that have unique meanings in TEAM are capitalized in the text.

## 3. Major Components and Regulatory Function of the GI Barrier

**Figure 1:**
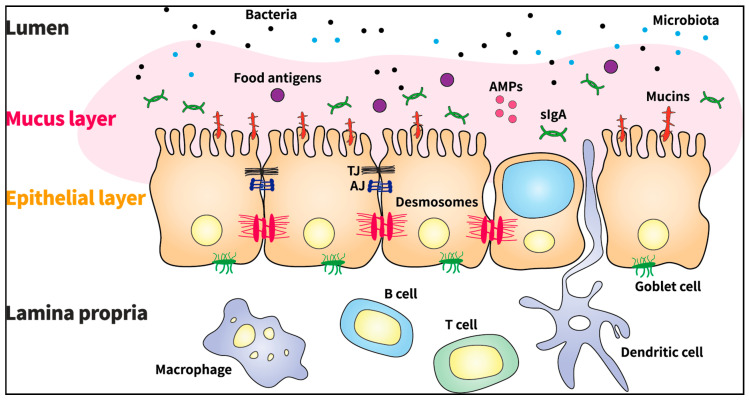
Components of the gastrointestinal barrier. AMPs: antimicrobial peptides; TJ: tight junction; AJ: adherens junction; slgA: secretory immunoglobulin A.

It is widely accepted that with respect to its association with innate immunity, the GI barrier acts as a semipermeable structure that allows the selective absorption of nutrients and facilitates immune sensing, whereas it restricts the entry of pathogens such as viruses, bacteria, prions, and fungi [1]. The barrier is composed of a mucus layer, an epithelial layer, and the lamina propria (Figure 1). The mucus layer is the first chemical defense barrier between external molecules and intestinal epithelial cells (IECs) [1,6]. The thick mucus layer is composed of mucins (MUC2, MUC5AC, MUC5B, and MUC6), which are highly glycosylated polymeric proteins, as well as other molecules secreted by goblet cells [6,7]. Defects in mucin gel assembly and production are related to GI inflammation and may lead to the development of several GI diseases such as spontaneous colitis [2,6].

The epithelial layer, present beneath the mucus layer, primarily prevents the translocation of pathogens into the blood circulation and maintains the integrity of the intestinal barrier. This layer is formed of a single layer of IECs with a rapid turnover rate. The lifespan of IECs is approximately 4–5 days. IECs contribute to the homeostasis of the layer, integrate the positive and negative interactions with the microbiota, and signal the immune cells to accommodate the microbiota [6]. Different types of specialized epithelial cells include absorptive enterocytes, Paneth cells, goblet cells, endocytes, and microfold cells [1,7]. The epithelial layers are interconnected via three adhesive complexes: desmosomes, adherens junctions (AJs), and tight junctions (TJs), which regulate the transportation of molecules between IECs [6,8]. The AJs are located below the TJs and form strong connections with desmosomes (also known as a macula adherens) to maintain epithelial integrity [1]. TJs are protein complexes formed from integral transmembrane proteins, including occludins, claudins, junctional adhesion molecules (JAMs), tricellulin, zonula occludens (ZO), peripheral membrane proteins, and regulatory proteins [9]. The integrity of TJs is dynamically regulated by the arrangement of actin and the interaction between transmembrane and peripheral membrane proteins [10]. The TJ complexes open and seal the barrier in the event of injury or upon receiving signals to enhance or modulate intestinal barrier homeostasis, and thereby form a highly dynamic entity [6]. AJs and TJs are attached to a dense ring of peri-junctional actin and myosin, which facilitates regulation of the junctions via the cytoskeleton [1,11]. In the event of intestinal injury or pathogen exposure, the pathogens are trapped in the intestinal mucus layer, antimicrobial peptides (AMPs) are released from Paneth cells, and the proinflammatory cytokines MCP-1, TNF-α, IL-18, and IL-6 are secreted by epithelial cells. In contrast, in the event of exposure to commensal gut bacteria, anti-inflammatory cytokines such as IL-10 are secreted by regulatory T cells, and the macrophages function as immune-regulatory cells to prevent excessive inflammation [9].

The lamina propria, which is a thin layer of connective tissue, plays a vital role in amicable communication between the immune cells and the gut microbiome. This layer contains T cells, B cells (secretory immunoglobulin A-producing plasma cells), stromal cells, and antigen-presenting cells such as macrophages and dendritic cells, which are components of the adaptive and innate immune systems [6]. The immune system is equipped with receptors named pattern recognition receptors (PRRs), such as Toll-like receptors (TLRs), for pathogen detection. These receptors are primarily expressed by macrophages and dendritic cells [9].

Collectively, the GI barrier, the first defense boundary that is composed of the mucus layer, epithelial layer, and lamina propria, plays a pivotal role in protecting the host against pathogens via intricate mechanisms.

## 4. Detrimental Effects of GI Barrier Dysfunction and Related Diseases

The term “GI barrier” is currently used to refer to the mucus layer or the underlying mucosal immune system [8]. Mucus layer damage is characterized by reduced thickness and altered mucus composition [10]. MUC2-knockout mice were shown to develop spontaneous colitis, which indicates the importance of the mucus layer [8]. However, the significant role played by this barrier in trans-mucosal water or solute flux is not attributed to the mucus layer, but to the epithelial monolayer, which is the key contributor to mucosal barrier function [8]. The IECs present in the epithelial layer, a highly regulated barrier controlling the entry of antigens for maintaining host health, exhibit a rapid turnover rate, which can jeopardize the integrity of the barrier. Specifically, failure of the rapid turnover mechanism can contribute to severe barrier defects, which may also lead to excessive intestinal inflammation and antigen invasion. Epithelial homeostasis is necessary for uninterrupted proliferation and maintenance of the delicate balance between progenitor cell proliferation and IEC apoptosis, which helps maintain the integrity of the barrier effectively [7,12].

Meanwhile, a wide range of factors may promote the loss of GI barrier integrity due to altered microbiota. These include direct or indirect factors arising from genetic susceptibility, diet (including high-fat diet and Western diet), toxic substances, pathogens, intake of drugs such as antibiotics, steroids, nonsteroidal anti-inflammatory drugs (NSAIDs), and acute or chronic stress [6]. Loss of barrier integrity increases permeability, which stimulates classic hypersensitivity responses to foods as well as to components of the normal intestinal microbiota. Under these conditions, bacterial endotoxins, cell wall polymers, and dietary gluten may induce the “non-specific” activation of inflammatory pathways via complement proteins and cytokines [13]. Overgrowth of Gram-negative bacteria may occur owing to increased permeability of the GI wall. These bacteria produce numerous endotoxins that can cause endotoxemia [14]. A previous in vivo study reported that autoimmune disorders may be attributed to chronic low-grade endotoxemia and imbalanced gut flora [15]. The disruption of normal GI barrier function due to endotoxemia is a crucial step in the pathogenesis of various diseases. The following diseases are commonly associated with increased GI barrier permeability.

### 4.1. Inflammatory Bowel Disease (IBD)

IBD is characterized by the onset of multiple conditions in which patients with increased paracellular permeability suffer from severe GI inflammation [9,16]. Ongoing GI symptoms consistent with Functional gastrointestinal disorders (FGIDs) are prevalent in IBD. Ulcerative colitis (UC) and Crohn’s disease (CD), the two major forms of IBD, are caused by intricate interactions among genetic, environmental, and immuno-regulatory factors [17]. In extreme cases of IBD, the production of multiple proinflammatory cytokines occurs in tandem with changes in the epithelial barrier function [2]. For instance, a recent study has shown that patients with UC or CD generally display epithelial barrier defects, especially TJ abnormalities [16]. The abnormal function of the GI barrier alone is sufficient to cause IBD, as shown in a previous study [7]. In addition, in patients with UC, the number of goblet cells directly participating in mucus secretion tends to decrease and the expression of β-defensins and AMPs is induced [18]. Conversely, patients with ileal CD have relatively low abundance of Paneth cells that produce α-defensins and additional AMPs such as lysozymes and secretory phospholipase A_2_ [16,19]. The expression of different defensins in patients with UC and CD may be attributed to differences between the major pathogenic factors influencing UC and CD [16]. This may be related to the fact that while inflammation is limited to the mucosa in UC, CD is characterized by transmural inflammation [20].

Gut microbiome dysbiosis, which could disturb the epithelial barrier function, is also associated with IBD [7]. A candidate gene encoding organic cation transporter 1 (OCTN1) was suggested as a susceptibility locus for CD [21], whereas epithelial-associated loci, such as CDH1, LAMB1, and HNF4A genes, were linked to UC susceptibility in a recent genome-wide study [22]. However, genetic variations exert a weaker effect on IBD development compared to environmental factors such as NSAID intake, antibiotic exposure, oral contraceptive use, and vitamin D deficiency, among others [2,23].

### 4.2. Celiac Disease

Celiac disease is a multisystem autoimmune disorder of the small intestine [2,24]. Accumulating evidence has indicated that celiac disease is more strongly linked to adaptive immunity than innate immunity [24]. Celiac disease is induced by dietary gluten in genetically susceptible individuals who carry the DQ2 or DQ8 human leukocyte antigen haplotypes [25]. It is characterized by villous atrophy of the small intestinal mucosa, malabsorption, and abnormalities in the small intestine epithelium [2], along with impaired TJ structure and increased intestinal permeability [26,27]. It is hypothesized that when gliadin penetrates the intestinal epithelium to enter the lamina propria, an immune reaction may be triggered. Gliadin is considered to increase intestinal permeability by inducing the release of zonulin, which reduces intestinal barrier function by inducing TJ disassembly [2]. The levels of zonulin, an important regulator of TJ permeability, were found to be six-fold higher in the intestinal submucosa of patients with celiac disease compared to that in healthy controls [28]. Since celiac disease is associated with other comorbidities, such as liver disease, pancreatic disease, and cardiovascular disease, the management of this condition is of significant importance. Celiac disease is also linked to nutritional deficiencies, osteoporosis, growth inhibition, infertility, skin diseases, small bowel cancer, and symptoms of limited nutrient absorption due to inflammation of small intestinal villi [24].

### 4.3. Irritable Bowel Syndrome (IBS)

IBS, one of the FGIDs, is a common and chronic condition that affects the digestive system, causing symptoms such as stomach cramps, bloating, diarrhea, and constipation. However, all symptoms cannot be attributed to specific pathophysiological factors. Patients with IBS suffer from constant GI symptoms and usually complain of depressive tendencies and anxiety disorders as well as a reduced quality of life [29]. A “leaky gut” is considered to induce the mucosal immune response, allowing low-grade GI mucosal inflammation and alterations in the levels of pro- and anti-inflammatory cytokines [30,31]. Mucosal mast cells and T lymphocytes are frequently present at higher levels in patients with IBS than in healthy controls. In addition, patients with IBS exhibit increased intestinal permeability and altered mucosal cytokine production more frequently, whereas the number of enterochromaffin (EC) cells (present alongside the epithelium lining in the lumen of the GI tract), B cells, and B lymphocytes, or mucosal serotonin production is not altered significantly. Earlier studies have suggested that IBS is associated with compromised epithelial barrier integrity and alterations in immune cell populations [31].

Several pieces of evidence show that multiple factors including infection, genetic predisposition, and stress link intestinal permeability and FGIDs [32]. Specifically, infectious gastroenteritis caused by pathogens such as *Campylobacter jejuni* is related to increased GI permeability, and certain single-nucleotide polymorphisms (rs12597188 and rs10431923 in the CDH1 gene) have also been associated with gut permeability. Furthermore, a number of previous studies performed in animal models have indicated that stress contributes to increased colonic permeability.

### 4.4. Obesity

Obesity is associated with GI permeability. A low level of inflammation is a notable characteristic of various metabolic diseases, including diabetes mellitus and obesity [33]. Augmented gut permeability and higher levels of plasma endotoxin and proinflammatory cytokines, including IL-1β, IL-6, INF-γ, and TNF-α, released due to barrier dysfunction were observed in a genetically obese mice model compared to that in control mice [10]. Furthermore, impairment of barrier function due to alterations in TJs has been observed in in vivo models of high-fat diet-induced obesity and diabetes [33]. An in vivo study showed that a high-fat diet suppresses the levels of occludin, claudin-1, claudin-3, and JAM-1 in the small intestinal mucosa, which increases the levels of plasma TNF-α [34]. Therefore, diet-induced obesity is responsible for abnormalities in TJs and alterations in gut microbiota in mice. These changes induce endotoxemia and metabolic syndrome caused due to high intestinal permeability followed by elevated lipopolysaccharide (LPS) absorption [33]. Gut microbiota-derived LPSs have been shown to be involved in the initiation and progression of inflammation and metabolic diseases associated with obesity and insulin resistance, such as type 2 diabetes. When present at high levels in plasma, LPSs, which are cell wall constituents of Gram-negative bacteria, can induce metabolic endotoxemia [2]. Antibiotics can be used to reduce circulating LPS levels and gut permeability [10].

### 4.5. Nonalcoholic Steatohepatitis (NASH)

GI permeability is known to be related to obesity and metabolic diseases such diabetes mellitus, obesity, and NASH [34]. In NASH, the heightened inflammatory response, which is induced by lipotoxicity caused by the accumulation of lipids in hepatocytes, is associated with alterations in the gut–liver axis resulting from increased GI permeability. Typical features of NASH include innate immune activation, inflammation, and fat accumulation during lipid overloading in the liver. While the process by which liver cells accommodate the lipid load may lead to adaptation, it may also lead to the development of isolated hepatic steatosis or the induction of cell death via a variety of distinct molecular mechanisms [35]. Malfunctions in the GI barrier lead to an increase in the levels of LPS and other bacterial-derived compounds in portal blood, which promotes the activation of TLRs and other PRRs in the liver and induces local inflammatory and fibrogenic responses.

In addition to the diseases discussed above, rheumatoid arthritis [36], schizophrenia [37], certain types of cancer [38], nonalcoholic fatty liver disease (NAFLD) [39], acne [40], atopy [40], autism [41], Alzheimer’s disease (AD) [42], alcoholism [43], acquired immunodeficiency syndrome (AIDS) [44], coronary heart disease [45], chronic fatigue and immune dysfunction syndrome [46], chronic arthritis/pain treated with NSAIDs [47], cystic fibrosis [48], dermatitis herpetiformis [49], pancreatic dysfunction [50], psoriasis [51], urticaria [52], and others have been found to be associated with increased GI permeability (Table 1). Multiple health problems have been related to increased GI permeability; however, conditions such as autism, acne, and urticaria have only been linked hypothetically to GI barrier impairment, and these findings have not been established clearly.

Although most diseases are usually caused by immune system dysfunction, most of the diseases discussed herein, including the skin conditions mentioned above, are specifically associated with immune system function, autoimmune reactions, inflammation, and endotoxemia.

## 5. Medicinal Plants for Treating Increased GI Permeability

*Flos Lonicera* Thunb. originated from eastern Asia and is a traditional medicinal herb, flower part of the plant, extracted by boiled water and has been used for several diseases for a long time. In addition, fermentation has been recently used to boost the beneficial effect of that on health. *Flos Lonicera* Thunb. was reported to alleviate obesity, which is now recognized as a chronic inflammatory disease, as well as associated metabolic endotoxemia by regulating intestinal microbiota and permeability in an in vivo model of high-fat diet-induced obesity. As mentioned earlier, exposure to LPSs may be related to abnormalities in the GI barrier function, which may lead to an increase in GI permeability. In a previous in vitro study, *Flos Lonicera* treatment inhibited the expression of ZO-1, claudin-1, and proinflammatory cytokine genes and suppressed LPS-induced NO production. In addition, *Flos Lonicera* is known to possess multiple pharmacological properties, such as antioxidative, anti-inflammatory, antiviral, hepatoprotective, and antihypertensive properties [77].

*Cudrania tricuspidata* has been traditionally used to treat several diseases such as pneumonia, phthisis, influenza, etc., in Korea. According to a recent article, dysfunctions in TJs can lead to several skin diseases, including atopy. The water extracts of the leaf were reported to alleviate atopic dermatitis by upregulating both mRNA and protein expression of claudin-1, which enhanced the potential of TJs. Claudin-1 maintains the integrity of the paracellular barrier in epithelial cells; therefore, loss of claudin-1 increases susceptibility to pathogens and skin barrier dysfunction. The antibacterial, anti-inflammatory, and anticancer properties of the extract have also been demonstrated in previous studies. The primary components of the extract include chlorogenic acid, kaempferol, and quercetin [78]. Among these components, quercetin, a common flavonoid, was shown to enhance TJ barrier function in Caco-2 cells by promoting the assembly of ZO-2, occludin, and claudin-1 via inhibition of the delta isoform of protein kinase C [79]. Furthermore, a previous study elucidated the intestinal barrier-enhancing effect of kaempferol on TJ assembly, especially in intestinal Caco-2 cell monolayers [79,80]. The extracts also exhibit antioxidant properties, modulate enzyme activities, and bind to signaling molecules or nuclear receptors [81]. Lastly, chlorogenic acid present in *C. tricuspidata* extracts was shown to enhance intestinal barrier integrity by downregulating myosin light chain kinase expression and promoting the dynamic distribution of TJ proteins [82].

Olive tree (*Olea europaea* L.) leaves and fruits are composed of hydrophilic and lipophilic bioactives, including phenolic acids (which are potent antioxidants), flavonoids, and secoiridoids. The plant bioactives are well-known for their antibacterial and antiviral properties and the beneficial effect exerted on humoral immune responses and cytokine gene expression. Liehr et al. showed that olive oil bioactives effectively counteracted TNF-α-mediated GI barrier disruption and improved the integrity of the intestinal mucosa and Caco-2 cells. The olive oil extract was obtained by extraction of the solid fraction and purification with ethanol and dissolving in methanol (5 mg/mL) [83].

Berberine is one of the primary components of *Coptidis rhizome* and a naturally occurring isoquinoline alkaloid and is commonly used to treat diarrhea, gastroenteritis, and colitis. *Coptidis rhizome* is traditionally extracted by hot water as other traditional medicinal herbs are and a recent study indicated that the hydroalcoholic solvent (50% ethanol, 50% water) at 35 ℃ for 30 min is the best extraction condition for the efficacy of bioactive compounds such as berberine [84]. Berberine alleviates UC and CD by inhibiting proinflammatory cytokine (IFN-γ and TNF-α)-induced intestinal epithelial barrier disruption. Increased gut permeability in patients with UC leads to the activation of TLR4, which has been observed to be upregulated in IBD. Oxyberberine, a novel metabolite formed by the modification of berberine by gut microbiota, may suppress TLR4 protein expression, which helps regulate intestinal mucosal inflammation [85]. Furthermore, proinflammatory cytokines have been reported to suppress TJ proteins, including ZO-1, ZO-2, and occludin. Berberine was shown to suppress IFN-γ- and TNF-α-induced intestinal epithelial barrier malfunction. Berberine is also considered to possess anti-inflammatory, antibacterial, antiparasitic, antioxidant, antiapoptotic, and antitumor properties [86].

*Rhizoma Atractylodis Macrocephalae* (RAM) is a traditional Chinese herb widely used in the treatment of GI disorders and both boiled water extraction and fermentation is usually used to extract bioactive compounds. RAM reportedly exhibits antioxidant properties and prevents viral gastroenteritis by protecting GI mucosal cells against injury. The antioxidant activity of RAM has been shown to play a significant role in protecting the GI barrier from LPS insult. LPSs can induce inflammatory responses in IECs that can lead to increased epithelial permeability, which is commonly associated with multiple GI diseases, including food allergies, IBD, and IBS, among others [87].

With regard to traditional therapeutic phytochemicals for leaky gut syndrome, most of the previous literature was limited to in vivo and in vitro study designs; thus, well-designed clinical trials are required to verify the effects. Medicinal plants, which exert a beneficial effect in leaky gut syndrome, are known to exert common antioxidant and anti-inflammatory effects (Table 2).

## 6. What Is Phlegm Syndrome?

**Figure 2:**
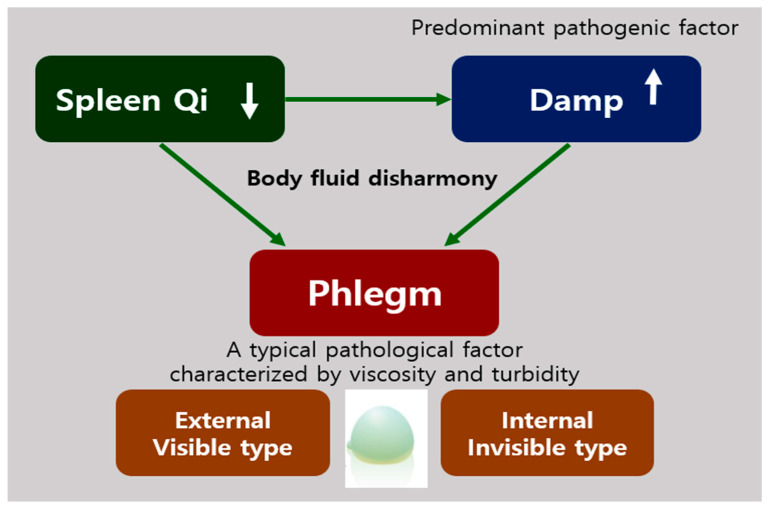
The etiology of phlegm in traditional East Asian medicine (TEAM). The weakness of Spleen Qi can lead to the generation of Damp, which causes body fluid disharmony occurring of Phlegm that are two types, external and internal types.

The spleen is an important organ in the human body that plays a significant role in red blood cell clearance and immune response. However, in TEAM, the functions of this organ are not limited to immunity and blood cell clearance, as it is considered to play an important role in the digestive system. According to the theories of TEAM, the spleen produces Qi and blood from digested foods and governs the transportation of water in the body [88]. Qi refers both to the refined nutritive substance that flows within the human body as well as its functional activities, having both biochemical and physiological features. Therefore, in TEAM, the spleen is usually referred to as a digestive organ, similar to the stomach and the small or large intestine, and Spleen–Stomach Qi is involved in the digestive process [3]. A weakness in the spleen Qi can lead to the generation of damp, which is one of the predominant physiological factors in addition to phlegm. Damp can also be generated due to environmental conditions, such as humid weather. Phlegm is formed when the body’s fluid metabolism (related to spleen Qi) is disrupted or when damp is not eliminated properly or remains in a stagnant state in the body for an extended period (Figure 2). Liver Qi stagnation also contributes to Phlegm production [3]. Phlegm is defined as a viscous and turbid pathological factor that is formed due to an imbalance in body fluid concentration and accumulates in certain parts of the body after condensation [3,66]. Phlegm can be released by the body (external or secretory form) in the form of sputum and nasal secretions from the respiratory tract, or it may be produced internally and may not be released (internal form) [89]. In particular, while the internal form of Phlegm has not been identified in studies, there have been studies on its nature and its potential for causing several diseases. Conversely, in Western medicine, phlegm is defined as a secretion produced in the airways, lungs, and upper respiratory tract during disease and inflammation. Phlegm contains mucus along with viruses, bacteria, debris, and shedded inflammatory cells, and sputum is the expectorated form of phlegm [90].

In TEAM, phlegm is considered to have a broader definition than that of sputum in Western medicine. Zhu Zhenheng, a traditional Chinese medicine (TCM) practitioner from the Yuan dynasty, stated that nine out of ten diseases were caused by Phlegm [89]. Additionally, strange afflictions have been attributed to Phlegm formation, since in its internal, nonsecretory form, Phlegm is considered to contribute to a variety of complex disorders [3]. Miscellaneous health problems may be attributed to Phlegm production, ranging from poor general health and digestive dysfunction to obesity, heart disease, cognitive impairment, autoimmune disorders, and tumors [3]. Based on the description of Phlegm in TEAM, its components do not seem to differ considerably from those of phlegm in Western medicine, as described above. In addition, Phlegm production is considered to be closely associated with inflammation in Western medicine [60]. Previous studies have shown that small molecules, including inflammatory factors and free radicals, might play a role in the interaction among Phlegm, stasis, and toxins [69]. Amyloids may be also considered to be related to Phlegm, since pathogenic amyloids are aggregated proteins that form fibrous deposits in plaques around cells and cause diseases such as amyloidosis, type 2 diabetes mellitus, and neurodegenerative disorders, including Parkinson’s disease, AD, and Huntington’s disease [91].

Nevertheless, since the above theory has not been validated, the precise composition of phlegm has to be determined in future research. The common symptoms of Phlegm syndrome are a muzzy feeling, heaviness in the head and body, palpitations, dizziness, fatigue, chest tightness, forgetfulness, irritability, bitter taste, dry mouth and throat, sticky mouth, general edema, swelling-induced pain in the head and eyes, and insomnia [89,92]. This description of the syndrome is specific to TEAM. The method used for describing a disease involves summarizing and studying the signs, symptoms, locations, causes, properties, and tendencies of the disease. In TEAM, a disease can be manifested as different syndromes in different patients; thus, identification of syndromes is a crucial part of the diagnostic process and treatment strategy in TEAM [93]. From a systems biology approach, in TEAM, a “syndrome” may be defined as the result of alterations in peptides or gene regulatory networks due to physiological dysfunction [94].

As an important pathogenic factor in several diseases, Phlegm is the causal agent of toxicity, stasis, and vessel injury. Phlegm syndrome has various names, such as Phlegm turbidity syndrome, Phlegm-Dampness syndrome, Phlegm stasis syndrome, Phlegm Heat syndrome, and Phlegm blood stasis, among others. Each type of Phlegm syndrome is slightly different from the others; therefore, the term “Phlegm syndrome” should be reclassified per standards to avoid confusion in future studies [93].

## 7. Diagnosis of Phlegm Syndrome

Phlegm syndrome is usually diagnosed by experienced physicians. In East Asian countries, including China, Korea, and Japan, pattern identification based on symptoms and signs is practiced as a unique diagnostic method in traditional medicine, which helps provide information for appropriate treatment [95]. This unique diagnostic system seems to have its roots in the philosophical difference between Western (conventional) and traditional (holistic) medicine, such as reductionism vs. holism. In the approach to pathogenesis adopted in TEAM, diseases are considered to affect the entire body rather than a specific part. The holistic approach adopted in traditional medicine has led to the development of individualized treatments for different patients with the same disease. This differs from modern personalized medicine; particularly, the method of evaluating a condition differs between both practices. Modern personalized medicine considers the genetic background and genotype of each individual, whereas individualized treatment in TEAM is conducted by observing the signs and symptoms exhibited by patients to define a phenotype that can guide treatment selection [95]. With respect to treatment, TEAM focuses on restoring physiological balance rather than curing a particular condition [96].

Since the 1980s, studies on the diagnostic criteria for syndromes, including Phlegm syndrome, based on TCM were initiated as part of projects on the scientific rediscovery and standardization of TCM. The process of distinguishing each syndrome involves the careful assessment of symptoms and signs using accepted criteria. The process is likely affected by the knowledge, clinical experience, academic background, and methods used by the physician, along with other factors [66]. Subsequently, the use of systems biology techniques allows thorough investigations based on a holistic approach. Using omics techniques, including genomics, transcriptomics, proteomics, and metabolomics, related studies have been actively conducted to identify biomarkers for the diagnosis of Phlegm syndrome in a standardized manner [97]. For instance, in a previous study on gene polymorphism analysis using the SNaPshot method, Han and Uyghur Chinese patients with hyperuricemia and Phlegm block were shown to harbor the rs2231137 allele C of the ABCG2 gene. In contrast, the rs2725220 allele G was associated with hyperuricemia in patients with nonPhlegm block [92]. In a Korean study on the relationship between gene polymorphisms and Phlegm syndrome, A-3826G and A-1766G polymorphisms in the UCP-1 gene known as obesity-related polymorphisms could be used as candidate genetic markers for the Phlegm-Dampness syndrome in stroke patients [98]. A self-reported Phlegm pattern questionnaire was developed using the Delphi method, reflecting the opinions of clinical experts in the context of TEAM. The questionnaire contained 25 items, each rated on a 7-point Likert scale, and could be administered as a validation study for healthy individuals [89]. Since this is a noninvasive and familiar approach, it can be easily applied in cross-sectional studies or descriptive surveys [65]. In addition to these methods for standardizing diagnosis, diagnostic systems based on images of the tongue [99,100] and tactile sensor systems for diagnosis based on pulse rate [101] have also been developed. However, despite the diverse efforts made, research on this subject is still at a preliminary stage, and reliable biomarkers for the accurate diagnosis of Phlegm syndrome are yet to be identified. The complexities involved in the interpretation of signs and symptoms in TEAM may lead to diagnostic errors [95]. Diagnostic technologies, diagnostic kits, or instruments such as endoscopy probes or computed tomography scanners that display visible or graphical test results should be utilized to convince patients of the severity of Phlegm syndrome and to perform accurate diagnosis.

There are multiple aspects to be considered before Phlegm syndrome can be diagnosed using scientific and standardized methods. These include identification of the components of invisible Phlegm, development of diagnostic equipment for Phlegm syndrome, and standardization of English terms.

## 8. Medicinal Plants for Treating Phlegm Syndrome

Tanrequing injection (TRQ, a traditional Chinese medicine formula containing Radix *Scutellariae*, bear bile powder, goral horn, *Flos Lonicerae*, and Fructus *Forsythiae*), along with phytochemicals such as baicalin, chlorogenic acid, ursodeoxycholic acid, and chenodeoxycholic acid, is widely used in TEAM for treating “Phlegm-Heat syndrome” and acute upper respiratory infections such as bronchitis and pneumonia [102,103]. The formula has been used as a capsule and an ample for injection. The capsule was made by extraction with 70% methanol for 30 min and filtration and the ample was manufactured by a pharmaceutical company in China in previous studies [102,104]. Modern pharmacological findings have shown that TRQ (approved by the China Food and Drug Administration) can alleviate inflammation in the respiratory system and reduce the high levels of mucus produced during bacterial and viral infections [103]. Furthermore, the effect of TRQ on acute exacerbation of chronic obstructive pulmonary disease (AECOPD) related to Phlegm-Heat syndrome was reported in an RCT [102]. Chronic obstructive pulmonary disease (COPD) is a common disease characterized by chronic inflammation of the respiratory tract with symptoms such as cough, dyspnea, lung paralysis, or lung distension. In TEAM, the five components of TRQ mentioned above are considered to reduce internal heat and promote internal toxin release and expectoration in Phlegm syndrome [102].

The Xuan Bai Cheng Qi formula also appears to be beneficial for AECOPD with Phlegm-Heat syndrome. The formulation consists of *Gypsum fibrosum* (gypsum), *Rheum officinale Baill* (rhubarb root and rhizome), *Armeniacae amarum* (apricot seed or kernel), and *Trichosanthes kirilowii* (trichosanthes peel). The levels of proinflammatory cytokines, including TNF-α, IL-4, IL-8, IL-1β, and IL-6, during COPD exacerbation were observed to be lower in the Xuan Bai Cheng Qi group than in the group that did not receive the treatment. The formula was provided as a granule manufactured by a pharmaceutical company in China via decocting the herbs together, extraction, and drying [105].

In TEAM, Alzheimer’s disease (AD) is considered to be caused by weak Kidney Essence and Phlegm stasis and is commonly treated by reinforcing Kidney Essence, dispelling Phlegm, and promoting mentality [57]. A previous study has demonstrated the function of Bushenhuatanyizhi gradule, which is composed of Radix *Polygoni Multiflori*, *Rhizoma Panacis Japonici*, *Rhizoma Acori Tatarinowii*, *Caulis Bambusae In Taeniam*, *Phizoma Pinelliae*, *Poria*, and Radix *Palygalae,* prepared by the Department of Pharmaceutical Preparation of Hubei Hospital of TCM, in the improvement of cognitive function and quality of daily life in patients with AD [57,106]. In TEAM, AD pathogenesis is considered to be associated with Phlegm Dampness and Turbidity, which leads to obstruction of various Orifices in the body. In an earlier study, weak Kidney Essence and Phlegm stasis were proposed as the primary contributors to AD pathogenesis, based on which Bushenhuatanyizhi was used to reinforce Kidney Essence, remove Phlegm, and promote mental therapy for AD [57].

Qingjian capsule, which is known to alleviate Phlegm Dampness-derived obesity, is composed of *Nelumbo nucifera* (Lotus leaf), *Typha angustifolia L.*, *Stephania tetrandra S. Moore*, *Benincasa his pida* (Thunb.) *Cogn*, *Astragalus membranaceus* (Fisch.) *Bunge*, *Cyperus rotundus L.*, *Semen Sinapis Albae*, *Atractylodes macrocephala Koidz*, *Alisma plantago-aquatica L.*, *Rheum palmatum L.*, and gold. Herbal medicines, including different phytochemicals, are known to dissolve Phlegm, eliminate Dampness, and replenish the spleen and moving Qi [70]. The English counterpart of the scientific name of each herb could not be determined.

Among the syndromes recognized in TEAM, early onset hypertension is considered to be related to the stagnation of Phlegm and blood stasis in the Meridians and hyperactivity of Liver Yang. Yinian Jiangya Yin, composed of 20 g of *Ramulus Uncariae cum Uncis*, 25 g of *Concha Haliotidis*, 20 g of *Plastrum Testudinis*, 10 g of *Rhizoma Pinelliae Praeparata*, 8 g of *Pericarpium Citri Reticulatae*, 12 g of *Fructus Aurantii*, 15 g of *Herba Leonuri*, 25 g of *Radix Achyranthis Bidentatae*, 20 g of *RamulusLoranthi*, and 20 g of *Radix Polygoni Multiflori*, is decocted with water into 200 mL a day and 100 mL of the decoction is orally taken twice a day. The decoction is known to calm the liver, nourish tendons, eliminate Phlegm, and allow the Meridians to flow smoothly; therefore, it is considered effective for lowering blood pressure in patients with early hypertension [107].

In TEAM, Phlegm is considered the basic pathogenic factor in hyperlipemia and causative agent in blood stasis. Danshen Jueming granules are known to regulate blood lipid levels and lipoprotein metabolism in senile hyperlipemia. The ingredients include 15 g of *Radix Pseudostellariae*, 20 g of *Radix Salviae Miltiorrhizae*, 15 g of *Semen Cassiae*, 15 g of *Fructus Crataegi*, 10 g of *Rhizoma Alismatis*, 10 g of *Pericarpium Citri Reticulatae*, and 10 g of *Hirudo* [108]. 

In TEAM, different diseases are treated using medicinal herbal mixtures enriched with phytochemicals that are commonly known to eliminate Phlegm (Table 3). While a variety of herbal treatments are available depending on symptoms, the treatment of inflammation using medicinal plants and phytochemicals is considerably similar to the treatment prescribed for leaky gut syndrome

## 9. The Relation among Phlegm Syndrome, Leaky Gut, and Inflammation

In TEAM, health problems related to disturbances in the Spleen–Stomach Qi or its transportation and transformation functions lead to the subsequent accumulation of Damp and Phlegm, which results in Qi stagnation, dyspepsia, and Turbid sputum development [113,114]. A study on pattern identification of syndromes in traditional Korean medicine (TKM) showed that the Phlegm-Dampness pattern can be identified by hindered Qi movement, turbidity, heaviness, stickiness, and downward-flowing properties [115]. The major clinical symptoms of Phlegm-Dampness syndrome include heavy body and limbs, abdominal distension, chest tightness, loss of appetite, red tongue with Yellow Greasy Coating (fur), and smooth pulse [116]. Excessive consumption of sweet or fat-rich foods or alcohol, or exposure to humidity for an extended period can cause Phlegm Dampness [73,117]. This was also confirmed in a previous in vivo study in which an animal model of Phlegm syndrome was made with high-fat forage [118,119]. Patients with Phlegm-Dampness syndrome are considered to be susceptible to multiple metabolic diseases [116]. This could be attributed to the fact that Phlegm tends to accumulate in different body parts, which might give rise to various diseases such as acne [58], AIDS [59], asthma [60], autism [62], obesity [73], type 2 diabetes mellitus [67,70,116], coronary artery disease [66], coronary heart disease [65], metabolic syndrome [73], NAFLD [72], hypertension [70], hyperlipidemia [69], bronchiectasis [63], stroke [74], AD [57], scrofula [75], and epilepsy [117] (Table 1). Table 1 lists the diseases associated with leaky gut and Phlegm syndromes, which are considerably similar. The process by which Phlegm, in its invisible or internal form, spreads, causes various diseases, and accumulates in different body parts is yet to be determined.

Although this is a novel hypothesis and it is almost impossible to compare related diseases between increased GI permeability and Phlegm syndrome due to the obscure mechanisms and symptoms, it is likely that Phlegm, one of the significant pathological factors in TEAM, with other pathogens may invade the blood stream via disruption of the GI barrier to occur various diseases. Furthermore, the Phlegm may have been named a long time ago in TEAM before the specific pathogens such as viruses, bacteria, etc., were discovered and may refer to all or some of the other pathogens for the related diseases. In fact, Zhu Zhenheng from the Yuan dynasty, between the mid-13th century and the mid-14th century, mentioned Phlegm, whereas bacteria and viruses were discovered by scientists such as Pasteur and Koch in the late 1800s. If one etiology that causes diseases due to increased GI barrier permeability is Phlegm, might the two syndromes share some parts of symptoms, related diseases, and mechanisms including inflammation reaction? Further research is required to figure out this issue and in order to contribute to the promotion of public health, modern medicine and CAM should be integrated in a mutually complementary relationship, and the theoretical basis for the diseases should be integrated in the future.

There are other barriers in our body such as the blood–brain barrier (BBB). The intestine can impact the BBB, which is an important protective barrier in the CNS and a semipermeable structure similar to the GI barrier [120]. It is composed of capillary endothelial cells, astrocytes, and pericytes. It forms a barrier between blood circulation and the brain and extracellular fluid in the CNS [121]. The BBB is one of the structures via which the microbiome interacts with the CNS. Gut flora can alter peripheral immune cells to enhance their interaction with the BBB, and cytokines and other immune-related molecules are released from peripheral sites when gut flora interact with the CNS via the BBB [122]. Dysfunctions in BBB integrity have been reported to contribute to the progression of neurodegenerative disorders such as AD, Parkinson’s disease, and traumatic brain injury [121]. If so, can Phlegm invade the blood stream and cause CNS-related diseases when the permeability of the BBB through the GI tract increases as well as the GI barrier? While the answer to this question awaits further research, it may appear that this is likely to be discovered as well if it is verified that the increased permeability of the GI barrier causes the spread of the Phlegm as a pathogen across the whole body, leading to various diseases.

The previous literature has reported that IBS, one of the FGIDs, is related to both leaky gut syndrome and Phlegm syndrome. A previous study in Israel indicated that many IBS patients have at least one comorbid somatic complaint [123]. In line with the Israel study, a previous Korean study also provided evidence that patients diagnosed with a Phlegm syndrome with GI symptoms complained of extra GI symptoms [124]. In terms of this, physicians practicing TKM in the Society of Phlegm Mass Syndrome reported that indigested toxic Phlegm could spread in the body, which is referred to as “Phlegm Mass” or “Damjeok” in Korean, translating to “accumulated Phlegm Mass” [125,126]. In addition, Phlegm is responsible for the generation of antigenic substances such as various viruses and bacteria, which can lead to various autoimmune diseases including Behcet’s disease, rheumatoid arthritis, and Crohn’s disease. Thus, this could be subsequently associated with inflammatory reaction. However, they argue this hypothesis through clinical features in which patients with autoimmune diseases are treated and alleviated with drugs for getting rid of Phlegm toxins. Furthermore, unfortunately, they have not yet reported reliable results via well-designed clinical trials in a scientific way, but just reported case reports [127] because the society has just started.

Chen et al. reported that Phlegm syndromes recognized in TCM could be uniquely related to inflammation. Specifically, inflammatory cytokines, Xin-Qi deficiency, and Xin-Yang deficiency were typically observed to be interrelated in patients with coronary artery disease exhibiting Phlegm Turbidity [128,129]. Furthermore, Cao et al. asserted that inflammatory biomarkers play an important role in distinguishing between Cold-Phlegm syndrome and Heat-Phlegm syndrome in patients with bronchial asthma [60]. Nevertheless, the association between inflammation and Phlegm syndrome has only been studied in a few diseases associated with Phlegm syndrome. For instance, a previous study showed that the rate of Phlegm Turbidity syndrome increased in groups expressing high levels of the inflammatory biomarkers HCRP and MMP9 [130].

## 10. Influence on Gut Microbiome Composition

The gut microbiome contributes to various metabolic pathways, including those related to digestion, bioactive molecule production, xenobiotic metabolism, and immune system development [131,132]. Vitamins, amino acids, short-chain fatty acids, and metabolites formed through the action of intestinal microbiota are vital for the TCA cycle, oxidative phosphorylation, glycolytic pathways, and amino acid and fatty acid metabolism. In addition, the intestinal microbiota affects the intestinal barrier and participate in a continuous two-way interaction between the CNS and the GI tract. A stable microbiota maintains homeostasis and works in conjunction with the mucus layer to provide protection against pathogenic bacteria [131,132].

In contrast to intestinal homeostasis, dysbiosis is characterized by imbalances in the commensal and pathogenic microbial populations as well as in antigen and metabolite production [132]. It is now known that dysbiosis is related to the pathogenesis of intestinal disorders such as IBD and CD as well as extraintestinal disorders such as allergy, asthma, metabolic syndrome, cardiovascular disease, and obesity [132,133]. For instance, reduction in the diversity of the *Firmicutes* population in fecal microbiota, especially a lower abundance of *Faecalibacterium prausnitzi*, is commonly observed in patients with CD. In the colon, several species of phylum *Firmicutes* ferment complex carbohydrates and produce butyrate, which has been reported to mediate the antitumor effects of dietary fiber on colorectal cancer [134], improve mucus secretion [135], and contribute to other protective effects of the epithelial barrier [2]. Similarly, *Escherichia coli* pathobionts that impede the epithelial barrier are frequently detected in patients with IBD [2].

Increased GI barrier permeability causes “atopobiosis” (translocation of bacteria) and promotes the release of toxic substances into the blood stream [42]. Microbiome composition exerts a significant impact on the properties of the mucus layer, including its permeability [131]. While it remains unknown whether alterations in the intestinal flora induces GI barrier dysfunction or increased barrier permeability leads to alterations in intestinal microbiota, or whether both occur simultaneously, it is clear that the two events influence each other via complex mechanisms. A recent study on small intestinal dysbiosis and FGID symptoms showed an inverse correlation between small intestinal permeability and microbial diversity [56,136]. The study also revealed an association between decreased microbial diversity and specific GI symptoms after a short-term switch from a diet with high-fiber content to one with low-fiber and high-simple sugar contents; this is likely similar to the cause of Phlegm Dampness [136]. To the best of our knowledge, no study has reported the association between Phlegm syndrome and gut microbiota or dysbiosis; however, certain studies have reported alterations in the gut microbiota in response to treatment with herbal medicine as per TCM. As proposed in the study, intestinal microbiota digest herbal treatment agents and release active small molecules that exert beneficial physiological effects [137]. Ginsenosides are the most well-known example. The gut flora mediates the deglycosylation of ginsenosides, and the products formed exert profound pharmacological effects [138]. To gain insights on Phlegm syndrome, well-designed research strategies should be adopted in future studies, as the gut microbiome and Phlegm is a relatively new area of research.

When it comes to the association of gut microbiota with inflammation, accumulated evidence exists, which is not the case for Phlegm and intestinal flora. In general, infection, injury, and antibiotic treatment can trigger inflammatory responses in the gut. It is known that tissue injury makes it difficult for the gut to maintain stable gut microbiota, which leads to gut dysbiosis [139,140]. In contrast, alterations in the composition of intestinal microbiota due to environmental and genetic factors promote the development of inflammatory disease [141,142].

Collectively, augmented GI barrier permeability is closely associated with Phlegm syndrome, inflammation, and intestinal microbiota. Phlegm syndrome is also related to GI barrier integrity and inflammation. However, evidence that supports the link between intestinal microbiota and Phlegm syndrome is scarce; hence, this topic warrants further research.

## 11. Discussion

**Figure 3:**
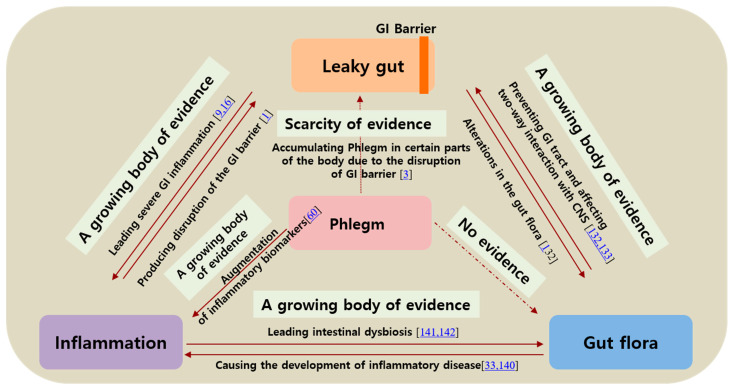
The quartet relationship between leaky gut-gut microbiota-inflammation-Phlegm.

In this review, we attempted to explore the relation between increased GI barrier permeability and Phlegm syndrome from complementary and alternative medicine by matching diseases and symptoms based on evidence from the published literature (Table 1). It is challenging to indicate the association between increased GI permeability and Phlegm without using measurement techniques or specific biomarkers because of the vagueness of symptoms and mechanisms. There are multiple limitations to this study, since we tried to evaluate the relationship between Phlegm and GI barrier permeability by comparing and contrasting concepts from modern medicine and TEAM. In addition, we discussed conditions related to invisible Phlegm, which is immeasurable using objective indicators. Additionally, the majority of studies on Phlegm syndrome have been conducted in China. However, in spite of the limitations and challenges encountered, our findings indicated the similarities between Phlegm syndrome and leaky gut syndrome with respect to associated diseases and suitable treatment agents, such as phytochemicals. The similarities may be attributed to the fact that both syndromes may share etiology and pathological mechanisms, which are strongly linked to inflammation. Phlegm is considered a broader subject area in TEAM, whereas leaky gut syndrome may be considered a subpart of a more complex condition. Studies on Phlegm syndrome have only commenced recently, whereas there is a growing body of evidence on the role of GI permeability (particularly intestinal permeability) in different diseases. This also makes it difficult to compare the two syndromes on the basis of evidence. The major areas of difference are as follows: (1) the number of studies reporting evidence, (2) quality of evidence, and (3) study design with method validation (Figure 3). Therefore, the hypothesis proposed herein should be investigated in future studies after the various contributing factors have been considered.

Since stringent search strategies, such as those used in systematic reviews, were not adopted in this study, all relevant articles may not have been considered. That said, systematic reviews conducted using search tools such as Medical Subject Headings, which is the list of terms provided by the National Library of Medicine used for indexing articles for PubMed, also do not cover all articles, and publication bias is never completely eliminated. Additionally, the authors’ perspective might contribute to the literature search process. We believe the theory proposed in this review should be investigated by adopting more objective and strategic approaches for this topic. Nevertheless, this review provides an alternative perspective for further research on Phlegm syndrome and increased intestinal permeability and the association between the two disorders, which could help identify effective treatments for the two syndromes related diseases such as the treatment of inflammation using medicinal herbs.

## 12. Conclusions

Although Phlegm syndrome and leaky gut syndrome seem to have some similarities with respect to related conditions and the suitability of phytocomplex treatment with anti-inflammatory and antioxidant activities, it is challenging to confirm that the two conditions are equivalent. According to TEAM, Phlegm syndrome is a broader area of study, and increased GI barrier permeability may be a subpart of this syndrome that is deeply associated with inflammatory reactions. The relationship among increased GI permeability, associated diseases, and intestinal microbiota is supported by a growing body of evidence. Based on the concepts defined in TEAM, Phlegm can accumulate in any body part and cause various disorders. However, research that correlates this condition with the leaky gut syndrome and gut microbial composition has only commenced recently. This study could provide novel insights for therapeutic targets and future research: (1) The GI barrier, which is closely associated with inflammation and endotoxemia, may be associated with Phlegm translocation in the body, which might be further associated with a wide range of disorders, including FGIDs. Therefore, biomarkers of inflammation or endotoxemia could be used in future studies on Phlegm syndrome and GI barrier permeability. (2) Phlegm of unknown composition may accumulate in the GI wall, which leads to a condition named “Phlegm Mass” (or “Damjeok” in Korean). (3) The complicated relationship among increased GI permeability, Phlegm, gut microbiota composition, inflammation, associated diseases, and treatments should be studied in future. (4) It will be necessary to adopt systems biology approaches along with omics techniques to explore the complexity of herbal medicines and phytochemicals on Phlegm syndrome and the underlying mechanism.

## Figures and Tables

**Table 1:** Comparison diseases and symptoms related to increased gastrointestinal barrier permeability and phlegm syndrome.

**Diseases related to Increased GI Barrier Permeability**	**Diseases Related to Phlegm Syndrome**
Alzheimer’s disease [42] Acne [40] AIDS, HIV infection [44] Alcoholism [43] Atopy/Eczema [40,53] Autism [41] Cancers [38] Celiac disease [26] Chronic fatigue and immune dysfunction syndrome [46] Chronic arthritis/pain treated with NSAIDs [47] Coronary heart disease [45] Crohn’s disease [17] Cystic fibrosis [48] Dermatitis herpetiformis [49] Diabetes mellitus [33] Gastroenteritis [54] Inflammatory bowel disease [12] Irritable bowel syndrome [55,56] Nonalcoholic fatty liver disease [39] Obesity [33] Pancreatic dysfunction [50] Psoriasis [51] Rheumatoid arthritis [36] Schizophrenia [37] Urticaria [52] Ulcerative colitis [17]	Alzheimer’s disease (dementia) [57] Acne [58] AIDS, HIV infection [59] Asthma [60] Atopy [61] Autism [62] Bronchiectasis [63] Cancers [3] Celiac disease [64] Coronary heart disease [65] Coronary artery disease [66] Diabetes mellitus [67] Irritable bowel syndrome [64] Epilepsy [68] Hyperlipidemia [69] Hypertension [70] Metabolic syndrome [71] Nonalcoholic fatty liver disease [72] Obesity [73] Stroke [74] Scrofula [75] Schizophrenia [76]
**Symptoms Associated with Leaky Gut Syndrome**	**Symptoms Associated with Phlegm Syndrome**
Arthralgias Abdominal distension Abdominal pain Cognitive and memory deficits Diarrhea Fatigue and malaise Fevers of unknown origin Food intolerances Myalgias Poor exercise tolerance Skin rashes Shortness of breath	Bitter taste Tightness in Chest Dizziness Dry mouth and throat Fatigue Feeling muzzy and heaviness in the head and body Forgetfulness General edema Palpitations Insomnia Irritability Sticky mouth Swelling pain of head and eyes

**Table 2:** Medicinal plants for treating increased gastrointestinal barrier permeability.

Medicinal Plants (MPs)	Major Components ^1^	Design	Disease or Beneficial Effects on Health	Primary Mechanism of Action	References
*Flos Lonicera* Thunb	organic acids, flavonoids, iridoid glycosides, saponins	in vitro in vivo	obesity obesity related metabolic endotoxemia	regulating of intestinal microbiota and permeability	[77]
*Cudrania tricuspidata*	chlorogenic acid, flavonoids (kaempferol, quercetin)	in vitro in vitro in vivo	atopy the enhancement of tight junction capacity	upregulating both mRNA and protein expressions of claudin-1	[78,79,80,82]
*Olea europaea*	polyphenols, flavonoids, secoiridoids	in vivo	nonalcoholic steatohepatitis, nonalcoholic fatty liver disease	lowered extracellular signal-regulated kinase activation in hepatocytes	[83]
*Coptidis rhizome*	berberine	in vivo in vitro	ulcerative colitis and Crohn’s disease	suppression of MLCK-MLC phosphorylation signaling pathway	[85,86]
*Rhizoma Atractylodis Macrocephalae* (fermented by *Bacillus licheniformis*)	sesquiterpene (atractylon, atractylenolide I, II, III), sesquiterpenoid (atractyloside A)	in vitro	several gastrointestinal diseases including food allergies, inflammatory bowel disease, irritable bowel syndrome	protecting on IECs against LPS-insult	[87]

1 If the major components of Medicinal Plants (MPs) were not listed in previous studies, they were found by searching from Korean Medicine Convergence Research Information Center, available online: https://www.kmcric.com (accessed on 4 February 2021).

**Table 3:** Medicinal plants for treating phlegm syndrome.

Formula	Medicinal Plants	Major Components	Disease or beneficial Effects on Health	Primary Mechanism of Action	References
Tanrequing (TRQ)	Radix *Scutellariae*	flavonoids, lignin	Acute exacerbation of chronic obstructive pulmonary disease Phlegm-Heat syndrome	removing internal Heat, releasing internal toxins, and promoting expectoration of Phlegm	[102,103]
	bear bile powder	-			
	goral horn	-			
	*Flos Lonicerae*	iridoid, secoiridoid, phenolic, triterpene, triterpene			
	Fructus *Forsythiae*	phenolic, lignan			
Xuan Bai Cheng Qi	*Gypsum fibrosum* (gypsum)	calcium sulfate	Acute exacerbation of chronic obstructive pulmonary disease Phlegm-Heat syndrome	suppressing proinflammatory cytokines including TNF-α, IL-4, IL-8, IL-1β, and IL-6 detected during COPD exacerbation	[105]
	*Rheum officinale Baill* (rhubarb root and rhizome)	dianthrone glycoside, anthraquinone			
	*Armeniacae amarum* (apricot seed or kernel)	amygdalin, prunasin			
	*Trichosanthes kirilowii* (trichosanthes peel)	triterpene (karounidiol, 3-epidarounidiol, and bryonolol, among others.)			
Bushenhuatanyizhi	Radix *Polygoni Multiflori*	emodin, chrysophanol, phycion, rhein, chrysophanol anthrone, resveratrol, piceid, epicatechin	Alzheimer’s disease Phlegm-Dampness and Turbidity syndrome	reinforcing Kidney Essence, removing Phlegm, and promoting mental therapy	[57,106]
	*Rhizoma Panacis Japonici*	*P*. *japonicas* saponins (Chikusetsusaponin V, Pseudoginsenoside RT1, Chikusetsusaponin IV, Chikusetsusaponin Iva) [109]			
	*Rhizoma Acori Tatarinowii*	phenylpropanoids (β-asarone, α-asarone, tatarinoids B, isoacoramone), lignin (ligraminol D) [110]			
	*Caulis Bambusae In Taeniam*	phenylpropanoid (p-coumaric acid)			
	*Rhizoma Pinelliae*	phenolic (homogentisic acid, 3,4-dihydroxybenzaldehyde)			
	*Poria*	triterpenoids (pachymic acid, eburicoic acid, and tumulosic acid, among others)			
	Radix *Palygalae*	saponin (polygalasaponin, tenuifolin), triterpene (senegenin), xanthone (1,2,3,7-teteramethoxyxanthone, 6-hydroxy-1,2,3,7-tetramethoxyxanthone)			
Qingjian	*Nelumbo nucifera* *(Lotus Leaf)*	alkaloids (neferine, nuciferine) flavonoids (catechin, kaempferol, quercetin)	Obesity Phlegm-Dampness syndrome	dissolving Phlegm, removing Dampness, and replenishing spleen and moving Qi	[70]
	*Typha angustifolia L.*	saponins, flavonoids, coumarins			
	*Stephania tetrandra S.Moore*	alkaloids (tetrandrine and fangchinoline, among others), flavonoids (stephaflavone A, stephaflavone B), steroids (β-sitosterol, β-stigmasterol) [111]			
	*Benincasa his pida(Thunb.)Cogn*	triterpenes (alnusenol, multiflorenol, isomultiflorenol), sterols (lupeol, lupeol acetate, β-sitosterol), glycosides, saccharides, caretenes, β-sitosterin, tannins and uronic acid [112]			
	*Astragalus membranaceus (Fisch.) Bunge.*	isoflavonoids (astraisoflavan, formonetin, astrapterocarpan), saponins (astragaloside I, isoastragaloside)			
	*Cyperus rotundus L.*	sesquiterpene (cyperene, cyperol, α-cyperone, cyperotundone, cyperolone)			
	*Semen Sinapis Albae*	glucosinolate (sinalbin), myrosinase, sinapine, 4-hydroxybenzylamine, p-hydroxybenzyl isothiocyanate, choline, β- sitosterol [113]			
	*Atractylodes macrocephala Koidz.*	sesquiterpene (atractylon, atractylenolide I, atractylenolide II, atractylenolide III), sesquiterpenoid (atractyloside A)			
	*Alisma plantago-aquatica Linn.*	triterpenoid (alisol A, alisol A 240acetate, alisol B, alisol B 23-acetate), sesquiterpenoid (alismol)			
	*Rheum palmatum L.*	Dianthrone glycoside (sennoside A), dianthrone glycoside (sennoside A), anthraquinone (chrysophanol, emodin, aloe-emodin, rhein)			
	gold	-			
Yinian Jiangya Yin	*Ramulus Uncariae cum Uncis*	alkaloid (rhynchophylline, corynoxeine, isocorynoxeine, isorhynchophylline, geissochizine methyl ether)	Hypertension Phlegm and blood stasis syndrome	calming the liver, nourishing tendons, removing Phlegm, and clearing Meridians	[107]
	*Concha Haliotidis*	CaCo_3_			
	*Plastrum Testudinis*	(+)-4-cholesten-3-one, cholesterol miristate, sterol			
	*Rhizoma Pinelliae Praeparata*	phenolic (homogentisic acid, 3,4-dihydroxybenzaldehyde) The ingredients of *Pinellia ternata Breitenbach* were described.			
	*Pericarpium Citri Reticulatae*	flavonoids (hesperidin, neohesperidin, poncirin and naringin, among others), monoterpene			
	Fructus Aurantii	flavonoids (hesperidin, neohesperidin, poncirin, naringin), monoterpene			
	*Herba Leonuri*	alkaloids (leonurine, stachydrine), flavonoid (rutin), terpene (prehispanolone, leosibirin)			
	Radix *Achyranthis Bidentatae*	steroids (ecdysterone, inokosterone, ponasteroside A, rubrosterone)			
	*Ramulus Loranthi*	flavonoids (avicularin, quercitrin), terpene (oleanolic acid, a-amyrin, corianin)			
	Radix *Polygoni Multiflori*	emodin, chrysophanol, phycion, rhein, chrysophanol anthrone, resveratrol, piceid, epicatechin			
Danshen Jueming	Radix *Pseudostellariae*	palmitic acid, linoleic acid, glycenal 1-monolinolate, behenic acid, 2-minaline	Hyperlipemia Phlegm syndrome	regulating blood lipid and metabolism of lipoproteins in senile hyperlipemia	[108]
	Radix *Salviae Miltiorrhizae*	penolic (salvianolic acid B), diterpene (tanshinone I, tanshinone IIA, miltirone, cryptotanshinone)			
	*Semen Cassiae*	anthraquinone (chrysophanoll, physcion, emodin, obtusifolin, obtusin)			
	*Fructus Crataegi*	flavonoid (quercetin), pinnatifinoside A, B, C, crataegolic acid			
	*Rhizoma Alismatis*	triterpenoid (alisol A, alisol A 24-acetate, alisol B, alisol B 23-acetate, alismol), sesquiterpenoid (alismol)			
	*Pericarpium Citri Reticulatae*	flavonoids (hesperidin, neohesperidin, poncirin, and naringin, among others), monoterpene			
	*Hirudo*	-			

## Data Availability

The data presented in this study are available on request from the corresponding author.
